# Early Life Maternal Separation and Maternal Behaviour Modulate Acoustic Characteristics of Rat Pup Ultrasonic Vocalizations

**DOI:** 10.1038/s41598-019-54800-z

**Published:** 2019-12-12

**Authors:** Jasmine H. Kaidbey, Manon Ranger, Michael M. Myers, Muhammad Anwar, Robert J. Ludwig, Alexandra M. Schulz, Joseph L. Barone, Jacek Kolacz, Martha G. Welch

**Affiliations:** 1 Department of Psychiatry, Developmental Neuroscience, Columbia University Irving Medical Centre, New York, NY 10032 USA; 2 School of Nursing, University of British Columbia, Vancouver, BC V6T 2B5 Canada; 3 Department of Paediatrics, Columbia University Irving Medical Centre, New York, NY 10032 USA; 4 Traumatic Stress Research Consortium, Kinsey Institute, Indiana University, Bloomington, IN 47405 USA; 5 New York Medical College, Valhalla, NY 10595 USA

**Keywords:** Emotion, Social behaviour

## Abstract

Early separation of preterm infants from their mothers has adverse, long-term neurodevelopmental consequences. We investigated the effects of daily maternal separation (MS) of rat pups from postnatal days 2–10 (PND2–10) on neurobehavioural responses to brief isolation at PND12 compared with pups receiving controlled handling without MS. Ultrasonic vocalizations (USV) were measured at PND12 during two, 3-minute isolations occurring immediately before and after a 3-minute maternal reunion. There were no significant differences in acoustic characteristics between MS and control animals in the first isolation. However, in the second isolation, MS pups produced a greater proportion of high (~60 kHz) vs low (~40 kHz) frequency calls. During this isolation, control pups made longer and louder low frequency calls compared to the first isolation, whereas MS pups did the opposite. Maternal behaviour of control and MS mothers modulated pup acoustic characteristics in opposite directions; higher maternal care was associated with more low frequency calls in control pups but more high frequency calls in MS pups. We hypothesize that MS results in USV emission patterns reflective of a greater stress response to isolation. This translational model can be used to identify mechanisms and interventions that may be exploited to overcome the negative, long-term effects of MS.

## Introduction

Due to medical necessity, preterm infants are cared for in neonatal intensive care units (NICU), where they are separated from their mothers for the majority of each day. The Nurture Science Program at Columbia University (nurturescienceprogram.org) sought to ameliorate short- and long-term consequences of early birth by facilitating mother/infant emotional connection during their stay in the NICU with the Family Nurture Intervention (FNI). A randomized controlled trial compared infants receiving standard care alone with infants that received FNI plus standard care (ClinicalTrials.gov, NCT01439269). This trial has produced several important outcomes including evidence that FNI increased maternal sensitivity during normal care-giving activities toward the end of the NICU stay, and decreased symptoms of maternal anxiety and depression at 4 months infant corrected age^1,2^. Near term, FNI infants had increased high frequency electroencephalogram power, altered electroencephalogram-coherence, and advanced maturation of central and autonomic nervous system function^3–6^. At 18 months of age, FNI infants had improved cognitive and language scores on the Bayley, fewer attention problems on the Child Behavioural Check List, and decreased risk for socio-emotional problems as assessed by the Modified Checklist for Autism in Toddlers^7^. These results have prompted increased interest in understanding the mechanistic underpinnings by which early maternal contact and emotional connection support infant development. Toward this end, here we have studied maternal separation and mother/pup interactions in rats as modulators of infant behavioural responses to the challenge of brief separation from their mothers.

Rodent ultrasonic vocalizations (USVs) are used in ethological studies and translational research of neuropsychiatric disorders as an index of social communication and emotion^8^. Extensive and diverse paradigms suggest an association between USVs and affective states depending on the frequency of the USV calls^9–13^. Low frequency (22-kHz) calls that appear in juveniles and remain throughout adulthood are markers of aversion, as they are emitted upon painful procedures, social defeat, and male-male aggression. Whereas, higher frequency calls (50-kHz) are emitted during rewarding interactions, like mating, play fighting, and even tickling^13^. Previous findings suggest that these associations of frequency and affect may be too crude since male rats also emit 22-kHz calls following ejaculation^14^. Associations between USV frequency and affect may not pertain to pre-weaning rat pups for two main reasons. First, in contrast to adults, pups mostly vocalize in the absence, not the presence, of conspecifics^8^. As a result, most studies in the neonatal period only report isolation-induced USVs, as these can be reliably elicited. Second, while it was initially believed that pup vocalizations were limited to 40-kHz calls^8^, later studies showed that isolation calls range from 5–120 kHz, but seem to concentrate around two frequency ranges, 35–40 kHz and 50–70 kHz^8,9,15^. USV calls can be additionally discriminated by duration: on average, low frequency calls are longer and higher frequency calls are shorter^9,10,16^. In a small study in Long Evans rat pups, the number of low frequency (40-kHz) calls increased upon a single exposure to rough maternal behaviour, while high frequency (66-kHz) calls were unaffected^9^. The effects of maternal behaviour over longer periods of time, or throughout development, on these two types of USVs are unknown. With the exception of studies using pups that were selectively bred for low and high call rates, it is unknown whether natural variation in maternal care during development shapes vocalization production^17^. Given the wealth of studies showing that maternal care mediates social behaviour and physiological outcomes, it is plausible that maternal care could impact USV production^18–23^. However, variation in natural maternal care is rarely observed in studies with USV outcomes, and thus the possibility that it may impact USV is important to consider^24^.

We studied the effects of repeated maternal separation (MS), a model of low maternal care and early-life stress, on USV acoustic properties. Pups having MS from postnatal days 2–10 (PND2–10) were compared with those handled to the same degree but without MS during this period. First, we investigated how daily MS in the neonatal period affected vocalizations at PND12, compared to normally reared, but control-handled pups. Second, we assessed whether natural variation in maternal care during early development shaped acoustic characteristics of USV production, with the hypothesis that more maternal care would mitigate changes in USV associated with MS.

## Results

### Maternal separation alters acoustic characteristics of ultrasonic vocalizations at postnatal day 12

**Figure 1:**
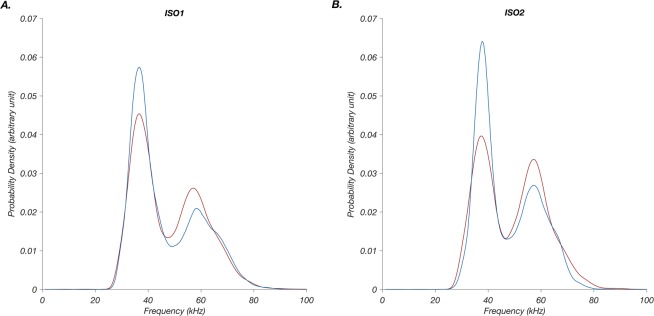
Collapsing across all ultrasonic vocalizations, maternal separation (MS) pups are more likely to emit higher peak frequency calls than control. (**A**) In the first isolation (ISO1), MS pups were more likely to call at higher frequencies (red trace), and control (blue trace) were more likely to call at lower frequencies. (**B**) This pattern became more pronounced in the second isolation (ISO2). Two-sample Kolmogorov-Smirnov tests confirmed that MS and control distributions are significantly different in both isolation periods [ISO1: *D* = 0.07, *p* < 0.0001, ISO2: *D* = 0.1, *p* < 0.0001]. Peak frequency means (±SEM) were similar in both groups and in both isolations: ISO1, 38 ± 0.07 kHz (n = 3,536 calls) and 60 ± 0.1 kHz (n = 2,997 calls) in MS vs 37 ± 0.05 kHz (n = 4,110 calls) and 61 ± 0.1 kHz (n = 2,228 calls) in control; in ISO2, 38 ± 0.05 kHz (n = 6,124 calls) and 59 ± 0.08 kHz (n = 7,356 calls) in MS vs 38 ± 0.04 kHz (n = 5,972 calls) and 59 ± 0.08 kHz (n = 3,629 calls) in control.

Ultrasonic vocalization (USV) call frequencies were first visualized using probability density functions. In the first isolation (ISO1), two distinct call frequencies were identified: one ~38 kHz and a second ~60 kHz (Fig. 1A). Both control and maternal separation (MS) pups were more likely to emit low frequency calls. Inspection of the probability density functions in the second isolation (ISO2), suggested that control pups distributed their call types similarly to ISO1 whereas MS pups shifted their distribution of calls to higher frequencies (Fig. 1B). Two-sample Kolmogorov-Smirnov tests confirmed that the distributions of vocalizations of MS and control pups are significantly different in both isolations (*p*-values < 0.0001).

**Figure 2:**
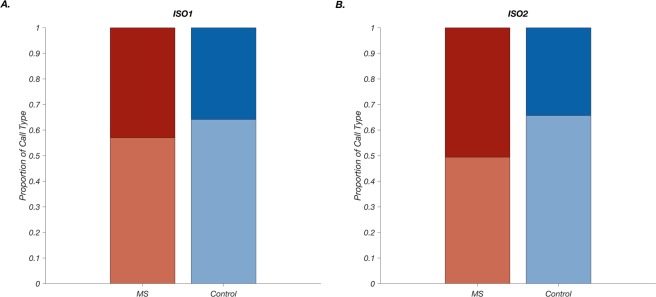
Maternal separation (MS) pups produce more high frequency calls and adjust low: high call frequency ratio in response to disrupted maternal reunion. (**A**) In the first isolation (ISO1), MS pups’ high frequency call proportion had a mean±SEM of 0.43 ± 0.04 (dark red) compared to 0.36 ± 0.06 in control (dark blue). (**B**) In the second isolation (ISO2), MS pups’ high frequency call proportion increased to 0.51 ± 0.04 (dark red) whereas control pups’ high frequency call proportion stayed about the same, 0.34 ± 0.04 (dark blue) (2-way repeated measure ANOVA; group: *F*_(1,26)_ = 4.09, *p* = 0.054; isolation: *F*_(1,26)_ = 1.64, *p* = 0.21, group x isolation interaction: *F*(_1,26)_ = 3.69, *p* = 0.066).

**Figure 3:**
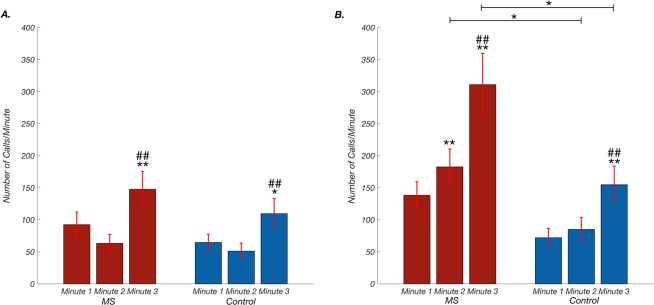
High frequency calling increases by minute during each isolation period. (**A**) In the first isolation (ISO1), there was a significant effect of time in high frequency call number production, but no significant effect of group or interaction (2-way repeated measure ANOVA; group: *F*_(1,26)_ = 1.06, *p* = 0.31; minute: *F*_(2,52)_ = 31.37, *p* < 0.0001; group x minute interaction: *F*_(2,52)_ = 0.99, *p* = 0.38). Maternal separation (MS) pups and control pups increased their high frequency calling between minutes 1 and 3 (post hoc *t* with Bonferroni correction; MS: *t*(13) = 4.37, p < 0.005, *d* = 0.624, control: *t*(13) = 3.76, *p* < 0.01, *d* = 0.612; and minutes 2 and 3 (MS: *t*(13) = 5.19, *p* < 0.001, d = 1.03, con*t*rol: *t*(13) = 4.12 *p* < 0.005, *d* = 1.23). B) In ISO2, all main effects were significant (group: *F*_(1,26)_ = 7.97, *p* = 0.009; minute: *F*_(2,52)_ = 48.08, *p* < 0.0001, group x minute interaction *F*_(2,52)_ = 5.57, *p* = 0.0064). The time points of significance were the same as in ISO1 (post hoc test with Bonferroni correction; minute 1 to 3: MS: *t*(13) = 5.75, *p* = 0.0002, *d* = 1.23, control: *t*(13) = 5.20, *p* = 0.0005, *d* = 0.961; minute 2 to 3: MS: *t*(13) = 5.46, *p* = 0.0003, *d* = 0.862, control: *t*(13) = 4.80, *p = *0.001, *d* = 0.907); in addition, MS were significantly more likely to increase high frequency calls from minute 1 to 2 (*t*(13) = 2.98, *p* = 0.032, *d* = 0.478). Post hoc tests showed group differences were not significant at minute 1 (*t*(13) = 2.71, *p* = 0.05, *d* = 0.971), but they were significant at minute 2 (*t*(13) = 2.78, *p* = 0.047, *d* = 1.09) and minute 3 (*t*(13) = 2.80, *p* = 0.046, *d* = 1.04). *Indicates a comparison to minute 1 where *p* < 0.05, **indicates a comparison to minute 1 where *p* < 0.005, ## indicates a comparison to minute 2 where *p* < 0.005. Horizontal bar indicates a comparison between groups.

MS pups also had a larger proportion of high frequency calls in both isolations compared to control pups (Fig. 2). Next, we analysed call frequency patterns during each minute, to determine whether pups were making dynamic changes during the isolation periods. In ISO1, there were no significant differences between groups; however, within each group pups increased their high frequency calls significantly from the first minute to the last (mean high frequency call number ± SEM, minute 1- MS: 92 ± 20, control: 65 ± 13; minute 2- MS: 63 ± 14, control: 51 ± 12; minute 3- MS: 148 ± 28, control:110 ± 24, Fig. 3A). This pattern continued in ISO2. Additionally, group differences became significant in ISO2: MS pups made a greater number of high frequency calls compared to control pups during the first and last minute (mean high frequency call number ± SEM: minute 1- MS: 138 ± 2, control: 72 ± 15; minute 2: MS: 183 ± 28, control: 81 ± 19; Minute 3- MS: 311 ± 49, control:155 ± 29, Fig. 3B].

**Figure 4:**
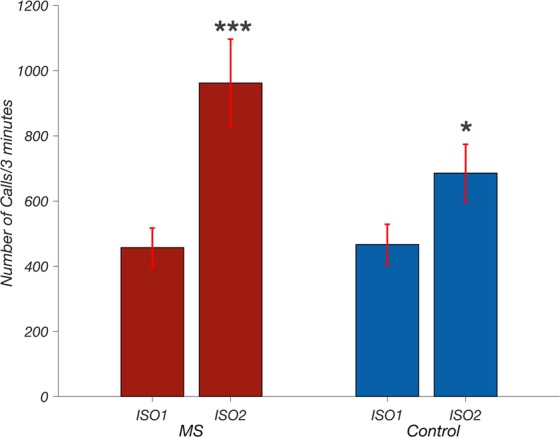
Call number by isolation and change score. In the first isolation (ISO1) the call number emitted by maternal separation (MS) pups was similar to control pups (mean ± SEM); MS: 467 ± 62 USVs/3 min, control: 457 ± 60 USVs/3 min. In the second isolation (ISO2) both groups emitted more calls; MS: 962 ± 130 USVs/3 min, control: 686 ± 60 USVs/3 min. Isolation period, but not group, had a significant effect on calling rate (2-way ANOVA with repeated measures- group: *F*_(1,26)_ = 1.52, *p* = 0.23, isolation: *F*_(1,26)_ = 29.79, *p* < 0.0001, group x isolation interaction: *F*_(1.26)_ = 3.77, *p* = 0.063). Furthermore, disrupting the reunion potentiated calls in both groups (Post hoc *t*-test with Bonferroni correction; MS: *t*(26) = 5.23, *p* < 0.0001, *d* = 1.26, control: *t*(26) = 2.49, *p* = 0.039, *d* = 0.81. *indicates a comparison between ISO1 where *p* < 0.05, ***p < 0.001indicates a comparison to ISO1 where *p* < 0.0005.

Analyses of calling rates showed additional differences between MS versus control pups’ USV acoustic qualities during the isolation that followed the brief maternal reunion. Both groups potentiated, i.e., increased their call rates significantly in ISO2, and while MS pups tended to potentiate more than control pups, the effect of group did not reach statistical significance (*p*-value for group by period interaction = 0.063, Fig. 4).

### Call duration, peak power, and peak frequency have conserved relationships

Duration and power (dB) of low frequency versus high frequency calls had distinct characteristics: low frequency calls were longer in duration and higher in power than high frequency calls. This pattern was consistent in both groups and in both isolations (ISO1 is shown in Table 1).

**Table 1:** Average duration and power for calls in ISO1.

Call Type	Group	Duration (ms)	Power (dB)
Low Frequency	Maternal separation	86 ± 8.2	78 ± 1.5
	Control	75 ± 3.9	77 ± 2.1
High Frequency	Maternal separation	9.4 ± 0.9	61 ± 1.0
	Control	10 ± 1.0	60 ± 1.8

Low frequency calls were significantly longer and louder than high frequency calls, in both groups (*p* < 0.00001).

**Figure 5:**
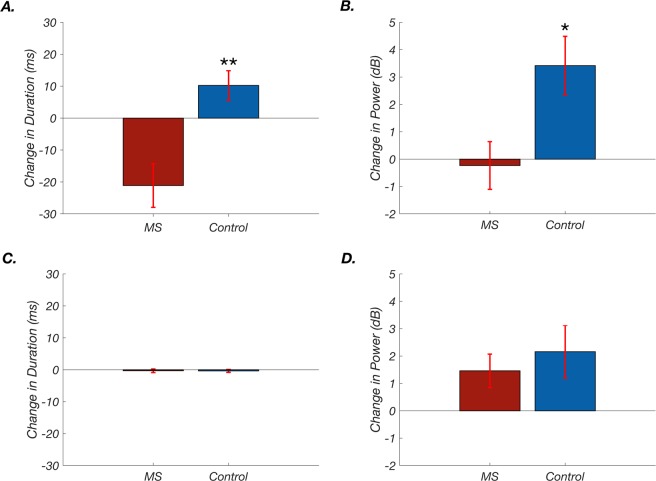
Maternal separation (MS) and control pups modulated duration and power of low frequency calls in opposite directions after a disrupted reunion with their dam. (**A**) Compared to the first isolation, in the second isolation MS pups emitted shorter low frequency calls whereas control pups had longer low frequency calls (mean change in call duration ± SEM: −21 ± 6.8 ms in MS vs 10 ± 4.6 ms in control; *t*(26) = 3.80,**p = 0.0008, *d* = 1.43). (**B**) MS pups also decreased the amplitude of low frequency calls whereas control pups increased them (mean change in power ± SEM: −0.23 ± 1.5 dB in MS vs 3.4 ± 2.1 dB in control, *t*(26) = 2.64, *p = 0.014, *d* = 0.997). (**C**) Neither group made significant changes in high frequency call durations; mean change in call duration ± SEM: −0.3 ± 0.6 ms in MS vs −0.3 ± 0.5 ms in control, *t*(26) = 0.056 *p* = 0.95, *d* = 0.0224. D) Similarly, high frequency changes in power were not significant; mean change in power ± SEM: 1.5 ± 0.61 dB in MS vs 2.2 ± 0.96 dB in control, *t*(26) = 0.61, *p* = 0.54, *d* = 0.232.

In ISO1, there were no group differences in average call duration or call power. In ISO2, however, pups in both groups significantly altered several acoustic characteristics compared to their ISO1 calls, and group differences emerged. While control pups responded to abrupt separation from their dam with longer low frequency calls, MS pups shortened their call lengths (Fig. 5a). Furthermore, the duration of low frequency calls was significantly shorter in the MS group (64 ± 8 milliseconds in MS vs 85 ± 6 milliseconds in control, *t(*26) = 2.01, *p* = 0.049, *d* = 0.780). In addition, control pups emitted somewhat louder low frequency calls in ISO2 and MS emitted slightly lower amplitude low frequency calls (Fig. 5b). Neither group significantly changed high frequency call duration (Fig. 5c) nor power (Fig. 5d).

### MS does not change maternal care at PND9-10

None of the maternal behaviours we assessed significantly changed from PND9 to PND10, so the average of Myers scores on PND9 and PND10 was used. There were no group differences in these maternal behaviour scores (MS: 0.52 ± 0.05, control: 0.44 ± 0.04, *t*(26) = 1.28, *p* = 0.21, *d* = 0.246), but we did find significant differences in length of licking and grooming (LG) bouts between MS and control dams, with control dams having longer bouts (38 ± 5.4 seconds in MS vs 55 ± 5.3 seconds in control, *t*(26) = 2.32*, p* = 0.029, *d* = 0.877).

### Maternal care on PND9-10 is correlated with several acoustic phenotypes on PND12

Higher Myers scores predicted more high frequency calls in ISO1 (Adjusted R^2^ = 0.11, *F*_(1,26)_ = 4.3, *p* < 0.05); Myers scores were not related to the number of low frequency calls (Adjusted R^2^ = −0.022, *F*_(1,26)_ = 0.42, *p* = 0.5), nor the proportion of either call type (Adjusted R^2^ = 0.0041, *F*_(1,26)_ = 1.1, *p* = 0.3). Given that there were no group differences in acoustic characteristics in ISO1, group designation was excluded in the statistical model investigating the relationship between maternal behaviour scores and acoustic characteristics for this isolation.

In ISO2, group designation was kept in our statistical model since most acoustic features were significantly different among the two groups. Myers maternal behaviour scores predicted high frequency call number in the same direction as found in ISO1: higher scores were associated with more high frequency calls (Adjusted R^2^ = 0.18, *F*_(1,26)_ = 4.0, *p* < 0.03; group beta = −205.5, *p* = 0.04; Myers score beta = 340.5, *p* = 0.2). Similar to the finding in ISO1, the model did not predict low frequency call number (Adjusted R^2^ = −0.047, *F*_(1,26)_ = 0.40, *p* = 0.7; group beta = −46.7, *p* = 0.6; Myers score beta = −217.5, *p* = 0.4).

Moving to low and high frequency call proportion, Myers maternal behaviour scores and group predicted 30% of their variance in ISO2 (Adjusted R^2^ = 0.30, *F*_(1,26)_ = 6.7, *p* < 0.005; group beta = 0.14, *p* = 0.019; Myers scores beta = −0.32, *p* = 0.059). Higher scores were associated with a lower proportion of low frequency calls and higher proportion of high frequency calls. However, when the analysis was split by group, Pearson product-moment correlations showed that these relationships only remained significant for the call proportions in the MS group (Proportion of Type 1 and 2 calls: MS: *r* = −0.54, *p* = 0.044; control: *r* = −0.18, *p* = 0.5).

LG bout duration was further explored and found to be a significant predictor of call frequency proportion in ISO2. A linear model with group and LG bout duration as variables significantly predicted higher proportion of low frequency calls (Adjusted R^2^ = 0.25, *F*_(1,26)_ = 5.58, *p* < 0.01); only group was a significant factor in this model (group beta = 0.13, *p* = 0.04, LG bout beta = 0.002, *p* = 0.16). This model predicted slightly more variance than when only group was included (25% vs 22%), but was less predictive than Myers scores, as described above. LG bout duration and Myers scores are highly correlated (*r* = 0.45, *p* = 0.01) and therefore were not included in the same model. Contrary to our findings with Myers maternal behaviour scores, LG bout duration is positively correlated with proportion of low frequency calls in control (*r* = 0.64, *p* = 0.01) but not the MS pups (*r* = −0.096, *p* = 0.7).

## Discussion

It is well established that repeated, early-life maternal separation (MS) has adverse consequences on both physiology and behaviour^25^. The present study is the first to extend the MS paradigm to acoustic characteristics of ultrasonic vocalizations (USVs) beyond a one-time MS behavioural test^26,27^. Our findings show that MS has repercussions on multiple acoustic features of USVs during a stressful test on postnatal day (PND) 12, when we acquired isolation-induced USVs. Specifically, daily MS during the first 10 days of life shifted the typical call frequency distribution found in normally reared pups to produce more high frequency calls after a disrupted reunion with their dam. We speculate that the shift to a higher frequency call type is indicative of greater experienced distress, or dysregulated stress reactivity. Furthermore, after the interrupted reunion, MS pups exhibited decreased power and duration of their low frequency calls, while control pups had the opposite response: another piece of evidence that these two groups deviate significantly in their USV response profiles. The observation that normally-reared pups have increased power and duration of call bouts after a reunion with their dam is consistent with a prior report^28^.

In an early seminal paper that manually classified USVs by sonographic structure, Brudzynski *et al*. found the percentage of high frequency calls increased with age from PND10 to PND17^15^. This raises the possibility that repeated separation prematurely accelerates distress communication, since the MS pups used more high frequency calls, although the use of littermates without correction in their experiment is a complicating factor.

The hypothesis that the USVs of MS pups may reflect increased distress is consistent with the perceptual advantage framework of the polyvagal hypothesis. This theory postulates that non-threatening social cues between mammalian conspecifics occur in the range of frequencies for which they have heightened sensitivity^29^. Rat audiogram studies reflect heightened sensitivity to calls near 40 kHz, which overlap with the low frequency call type described here^30–32^. The polyvagal hypothesis further postulates that cues occurring at frequencies outside the heightened sensitivity range, but still within perceptible frequencies, carry danger and distress information^33^. Examples exist in human infants as well as rats, and our data seems to support this hypothesis^34^.

Adhering to this framework, low frequency isolation calls can be viewed as a social cue that carries positive communicatory value, and high frequency calls as signal of distress. This would indicate that control pups communicate in a more adaptive fashion, as the majority of their USVs fall in the frequency band of perceptual advantage. The polyvagal hypothesis further proposes that acoustic information not only serves a communicatory function, but also as a window into the physiological state of the sender^29^. Slow exhalation, the respiratory process associated with expressive social vocalizations, enhances the impact of the vagus on the heart, promoting calm states^29^. Rat USVs occur during the expiratory phase of respiration, so low frequency vocalizations are associated with lower rates of respiration as a result of their longer duration^9^. Whether this indicates that control pups respond to isolation in a more adaptive manner, while simultaneously emitting USVs of higher communicatory value compared to MS pups, requires confirmation in future studies.

To our knowledge only two studies characterized acoustic characteristics of isolation USVs in normally reared Sprague Dawley pups into types distinguished by frequency^16,26^. In a comparative study of the three most widely used outbred strains (Wistar, Long Evans, and Sprague Dawley), Wöhr and Schwarting tested PND11 pups during a single ten minute isolation, then classified the calls into three clusters by frequency and frequency modulation^16^. Calls classified as Type 1 and 2 in their study resemble the low and high frequency calls described here, but they further classified low frequency calls (~39.6 kHz) into a third class that had large frequency modulation (called Type 3). In Sprague Dawley pups, isolation USVs were 87% type 1, 6% type 2, and 7% type 3. These percentages reflect significantly greater low frequency calls than we found in our control group. These differences may be due to differing criteria used to define calls, and the age of the pups studied. Moreover, they showed that many acoustic characteristics vary significantly by strain: peak frequencies, percent of call type used (i.e., our frequency proportions), call lengths, total time spent calling, and frequency modulation^3^. In two studies in Long Evans rats, pup isolation calls were also categorized into two types, low frequency calls averaged at 40 kHz and high frequency calls averaged around 50–66 kHz^9,10^. Contrasting these Long Evans findings with our own corroborates Wöhr and Schwarting’s finding that Sprague Dawley’s peak vocalization frequencies are slightly slower than Long Evans’. Thus, when comparing vocalization acoustic characteristics in rodents, it is important to consider strain and species since mouse and rat USV acoustics are also known to differ^12^. In one of the Long Evans studies, though the authors do not call their paradigm a potentiation test, Boulanger-Bertolus and colleagues collected USVs during two isolation periods with a maternal reunion in between^9^. They used this as a model of abusive caregivers since the reunion with the dam takes place in a small, bedding-free, novel cage which they suggest increases rough maternal interactions. During the isolation after the maternal reunion, pups potentiated the number of low frequency calls compared to those that had no reunion with their dam. Though an exact parallel cannot be drawn with our study since we did not have a group without a reunion with the dam, we did in fact find that both our groups emitted significantly more low frequency calls after the reunion. We did not, however, observe rough maternal interaction. Common interactions were sniffing, licking and grooming (both anogenital and whole body), and much less frequently, pup carry. Interestingly, preliminary analyses did indicate that pups who were carried – as opposed to just licked or not interacted with at all – emitted more low frequency calls, but this was not reported here because of the small number of events in which this type of interaction occurred.

In addition to findings on the acoustic characteristics of MS pup vocalizations, our behavioural observations shed light on whether the quantity or quality of maternal behaviour may mitigate separation stress and/or influence acoustic characteristics of USVs. While our home cage observations showed no differences in overall maternal behaviour scores between the MS and control dams, it is important to note that these measurements of maternal care were at PND9 and PND10 only. It is possible that differences in care earlier in life may have contributed to the acoustic differences in the pups. Peña *et al*. found that in a sample size of 34 litters, day and time of observation is a determinant in classifying a dam’s maternal behaviour accurately; using only light or only dark observations resulted in divergent classification of a dam’s licking and grooming level (i.e. low, medium, high). Similarly, using observations from a single postpartum day is also likely to result in divergent classifications. The implication of this temporal variation in care is that using a classification that does not accurately represent the dam results in misleading interpretation of data. In our study, we reported home-cage observations of the dam that compared the MS and control group at PND9 and PND10 as the type and timing of these observations have been validated as a method to establish peak dam-pup interaction up until PND8^24,35^. Several investigators have shown that maternal behaviour decreases over time^18,20,36^. In one study of Sprague Dawley dams, the group found that active maternal behaviour (a composite measure similar to Myers scores that additionally includes pup carry) decreased progressively from PND1 to PND5^20^. Another study, also in Sprague Dawley rats, found that while LG progressively decreased, ABN did not^18^. We suspect that these mixed results may be due to methodological differences in the timing of the observations. While Gatta *et al*. observed maternal behaviour for two hours a day (during the light cycle only), Clinton *et al*. used a computerized program that recorded their dam-pup interactions in 24-hour periods^18,20^. In addition to temporal differences in observations, many studies, including the present one, are limited by the characterization of the dam’s behaviour towards the entire litter without investigating whether that care is distributed equally among the littermates. It has been shown that on PND5 to PND8 Sprague Dawley dams exhibit sex differences in licking and grooming of pups, preferentially licking males, as well as within-sex littermate preferences where the tertile of pups that were most-licked within a litter consistently received more than twice as many licks as the least-licked tertile^37,38^. It may be worth noting that those studies were done with much larger litters, some more than double the size, than the present study.

With these methodological considerations in mind, we evaluated the influence of maternal care on acoustic characteristics of USVs, particularly on low/high call frequency distribution. The results were dependent upon the indicator used to reflect the dam’s maternal behaviour. In normally reared pups, more maternal care, proxied by licking and grooming (LG) bout duration, resulted in more low frequency calls, whereas LG bout duration was not related to call frequency distribution in the MS group. On the other hand, in the MS group, higher maternal behaviour, proxied by Myers maternal behaviour scores, correlated with elevated high frequency call number, whereas no significant relationship was present in the control group. Taken together, higher maternal care seems to be driving the two groups in different directions: in control pups it increases low frequency calls, whereas in MS pups it increases high frequency calls. This finding suggests that higher maternal behaviour alone may not mitigate MS stress. This is in line with Calming Cycle Theory, which postulates that in order for maternal care to have a calming effect on infant physiology there must be an emotional connection between the two^39–41^. Our study assessed maternal behaviour on PND9-10 only and did not assess the emotional connection between mothers and pups. While we believe that the rodent model used in this study will be helpful to identify physiological mechanisms underlying MS and reunion, future studies will attempt to assess mother/pup emotional connection upon reunion to determine whether it will have an effect on USV characteristics, as well as the corresponding underlying autonomic physiology. Continued analyses of USV acoustic characteristics is critical to deepen our understanding of whether low/high frequency call distribution can be used a as tool to understand the maternal pup dyad connection. Such findings may lead to effective naturalistic interventions to overcome the negative long-term effects of MS in humans.

## Methods

### Animals

Twenty-eight timed-pregnancy Sprague-Dawley females were received from Charles River (Wilmington, MA), and housed in a satellite colony room at the New York State Psychiatric Institute where they received routine husbandry. Animals were kept in 12-hour dark/light cycles with lights on at 7 am. They had ad libitum access to food and water, and corn cob bedding in polycarbonate terraria (40 × 20 × 24 cm^3^). Colony room temperature (21 °C) and humidity (40%) were regulated. All procedures were conducted strictly following our protocol approved by Columbia University Irving Medical Centre and New York State Psychiatric Institute’s Institutional Animal Care and Use Committees.

### Daily separation procedure for maternal separation (MS) group

On postnatal day (PND) 2, litters designated as MS (n = 14) were culled to eight pups (sex balanced whenever possible). From PND2 to PND10, the dam was removed from the home cage and placed in a novel cage with fresh bedding, food, and water in a separate room. Then, each pup was identified with a non-toxic marker, weighed, and placed in a novel cage with fresh bedding and cylindrical partitions to separate littermates. The cage was then placed in a dark, temperature-controlled incubator for three hours daily (10 am–1 pm) set at 34 °C (PND2-4), 33 °C (PND5-7), or 32 °C (PND8-10) to keep core body temperature stable^25,42^. Once the separation period ended, pups were returned to the home cage. Then, the dam was returned to the home cage, and the cage was returned to the housing room and left undisturbed until the following morning.

### Daily handling for control group

To more closely equalize handling of the two groups, both were handled daily. In the control group, handling started on PND1, at which point the litters (n = 14) were culled to eight pups (sex balanced whenever possible) and marked with a non-toxic coloured marker for identification purposes, weighed, and then returned to their home cage. This was repeated until PND12.

### Observation of maternal behaviour in MS and control groups

To allow group comparison of maternal behaviour, two 1-hour observation sessions of maternal behaviour were conducted on PND9 and PND10 between 3 and 5 pm. On these days, each litter was observed continuously for 60 minutes noting the start and stop time of the dam’s behaviour. The type and timing of the observation have been validated as a method to establish peak dam-pup interaction up until PND8^24,35,43^. All observations were done from recorded videos outside the housing room (by JHK) identifying naturally occurring behaviours previously described in detail^22^. Maternal behaviour composite scores (Myers maternal behaviour scores) were obtained by summing the time (in seconds) a dam spent arch nursing (both in high- and low-arch positions), licking and grooming, and in contact with her pups as this composite score has been shown to correlate to offspring physiology^22^. Then, Myers scores of individual dams were averaged within the group and used to compare maternal behaviour between groups. We also quantified bouts of licking and grooming (LG bouts), defined as the number of seconds of continuous licking without interruption, as an additional index of maternal behaviour.

### Potentiation of ultrasonic vocalizations (USVs) paradigm

Testing for maternal potentiation of USVs took place on PND12 based on an established method^44^. Thirty minutes before testing, the dam was removed from the home cage and taken to a separate room in a novel cage with fresh bedding, water, and food. The home cage with the pups was then placed in a novel room on a water recirculating heating pad. After this 30-minute period, each pup in succession was gently picked up from the home cage (test order was random), placed alone in a small (25 × 14 × 11.5 cm) novel clear polycarbonate rectangular box without bedding, and brought to the testing room (room temperature conditions, ~22 °C). Each pup was observed in the novel container during a first isolation of 3 minutes (ISO1), a reunion of 3 minutes with their dam (REUN), and a final 3-minute isolation (ISO2). During the isolation periods, USVs were recorded by an ultrasound sensitive microphone placed 2 inches above the test cage (sampling rate: 250 kHz, frequency range: 5–120 kHz) connected to a computer via the UltraSoundGate 116 USB audio device (Avisoft Bioacoustics, Glienicke, Germany). USV measurements during the isolation periods were displayed in real time with Avisoft Recorder (version 4.2, Avisoft Bioacoustics). Upon completion of ISO2, the pup was returned to the home cage, and the next pup was removed to go through the same testing sequence (each pup was placed in a clean, novel cage).

### Data analysis of potentiation and other acoustic characteristics of USVs

Raven Pro (Version 1.5, Cornell University Lab of Ornithology) was used for analysis of USV spectrograms. One male pup, chosen at random, was selected per litter for the current analysis. Males were selected since it has been shown that licking and grooming is preferentially given to males^37,38^. Given that our maternal behaviour observations did not distinguish among pups within the litter, we are limited to speculating that males are more likely to reflect effects of maternal care on USVs. A recent series of studies examining strain differences between the three most common inbred rat strains found that peak frequencies and the proportion of low/high frequency isolation-induced USVs were conserved between the sexes in unrelated male and female Sprague Dawley rat pups^16,45^.

The Band Limited Energy Detector of Raven Pro was used as a first pass to mark calls in the spectrograms. Then, each file was manually inspected to ensure accuracy of the detector’s selections and additional marks were added where necessary. A call was defined as a continuous, uninterrupted vocalization. The following measurements were determined for each identified call: peak frequency (kHz), peak power (dB), begin time (s), and end time (s). Peak power was defined as the point where maximum power occurs within the selection (i.e. call), and peak frequency was defined as the frequency at which peak power occurs within the selection. These data were subsequently imported into Excel.

For each pup, we determined; (1) the number and proportion of low frequency calls (number of calls <47.5 kHz divided by total number of calls) and high frequency calls (number of calls >47.5 kHz divided by total number of calls), (2) peak power and duration of calls in the low frequency subset of calls (<47.5 kHz), and (3) peak power and duration of calls in the high frequency subset (>47.5 kHz). These cut-offs were chosen post-hoc based on the distribution of the entire dataset of calls that showed two distinct clusters above and below 47.5 kHz (Fig. 1). Proportions of calls are reported in addition to call numbers to adjust for the varying number of total calls emitted by different pups. Data were imported into R for statistical analysis (R Core Team, 2015).

### Statistical analysis

#### USV acoustic characteristics

Two sample Kolmogorov-Smirnov tests were used to assess whether the distribution of total calls was distinct in the two groups. Two-way repeated measure Analysis of Variance (ANOVA) tests were used to compare groups (control, MS) when there was repeated data from the same pup: proportion of low and high frequency calls per isolation, and call rate per minute and per isolation; group and isolation period were used as the factors. Post-hoc *t*-tests with Bonferroni corrections were done when the ANOVA allowed. Unpaired, two-tailed *t*-tests were used to evaluate the change in call duration and the change in peak power of the two call types. *P*-values less than 0.05 were considered significant.

#### Maternal behaviour

Myers scores were calculated for each dam at PND9 and PND10. They were then averaged by group and groups were compared by an unpaired *t-*test. The same procedure was repeated for LG bout duration. *P*-values less than 0.05 were considered significant.

#### Effects of maternal behaviour on acoustic characteristics

Myers scores and LG bout lengths were used in Pearson’s product moment correlation models with call number, call frequency proportion, call duration, and call power, as well as linear regression models with the aforementioned acoustic characteristics. Analyses were done for both control and MS groups in both isolations separately. *P*-values less than 0.05 were considered significant.

## Data Availability

The datasets generated and analysed during the current study are available from the corresponding author on reasonable request.
